# The Effects of Catheter Ablation on Permanent Pacemakers and Implantable Cardiac Defibrillators

**DOI:** 10.19102/icrm.2017.080303

**Published:** 2017-03-15

**Authors:** Yousef H. Darrat, Gustavo X. Morales, Claude S. Elayi

**Affiliations:** ^1^Cardiology Department, Gill Heart Institute and VAMC, University of Kentucky, Lexington, KY

**Keywords:** Catheter ablation, implantable cardiac defibrillators, permanent pacemakers

## Abstract

Catheter ablation is a procedure that is frequently performed in patients with cardiac implantable electronic devices. Here, we review all of the potential interactions that can occur among patients undergoing catheter ablation while having implantable cardiac electronic devices, and discuss the precautionary measures to minimize such interactions.

## Introduction

**Figure 1:**
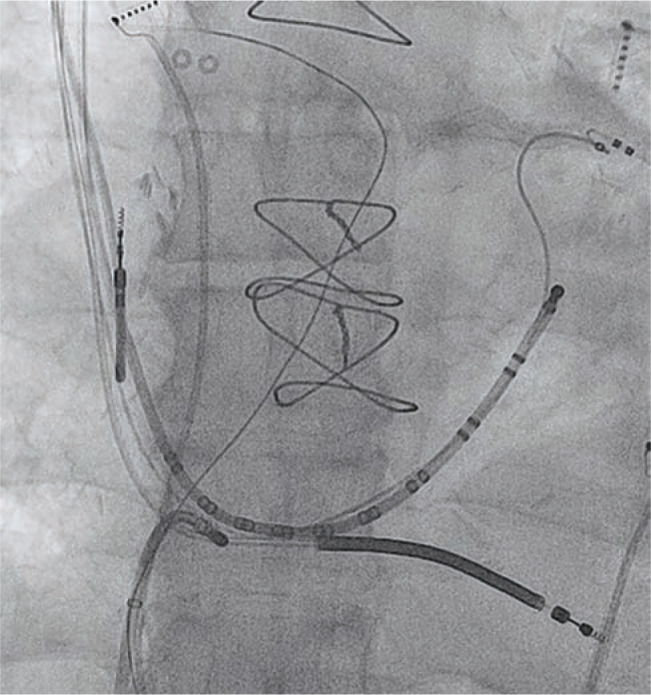
A patient with a biventricular implantable cardioverter-defibrillator undergoing cavotricuspid ablation for typical atrial flutter (left anterior oblique projection). Note the close contact between the right ventricular lead as it crosses the tricuspid valve and the radiofrequency ablation catheter.

**Figure 2:**
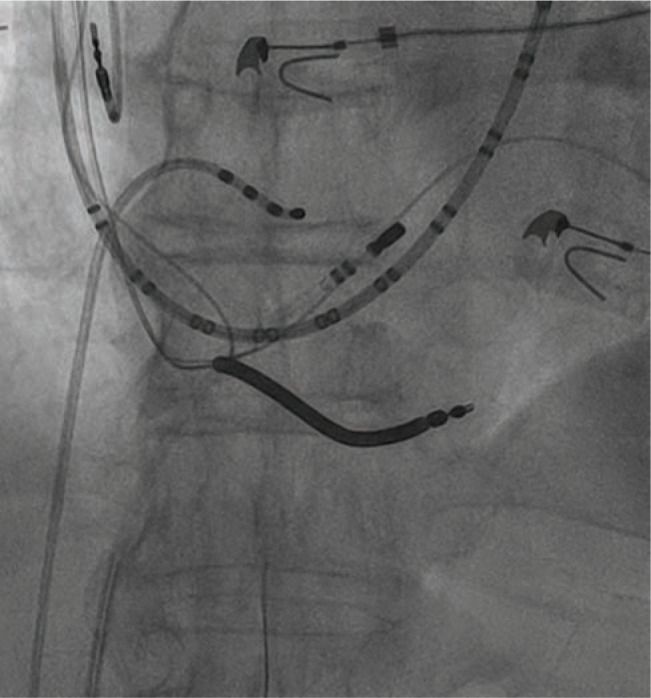
This patient with a biventricular implantable cardioverter-defibrillator also has a paroxysmal atrial tachycardia requiring ablation in the coronary sinus (left anterior oblique projection). The radiofrequency ablation catheter is inside the coronary sinus, in close contact with the left ventricular lead.

The use of cardiac implantable electronic devices (CIEDs) has witnessed a steady growth since the introduction of permanent pacemakers (PPMs) in the 1960s and implantable cardioverter-defibrillators (ICDs) in the 1980s.^1^ Meanwhile, over the past two decades, catheter-based ablation has emerged as an integral component of atrial and ventricular arrhythmia management.^2–4^ As a result, catheter ablations are becoming more often required in patients with previously implanted CIEDs. Cardiac electro-physiologists are, for instance, more likely to encounter patients with right ventricular or coronary sinus leads requiring a cavotriscupid ablation or ablation in the coronary sinus, respectively **(Figures 1 and 2)**. The Heart Rhythm Society and the American Society of Anesthesiologists have recommended avoiding direct contact between ablation catheters and the CIED systems.^5^ However, these consensuses are more based on precautionary measures, rather than on convincing clinical data, as clinical studies evaluating interactions between CIEDs and ablation catheters remain limited in number. Furthermore, prior studies can become quickly outdated as the technology associated with CIED and catheter ablation continues to rapidly evolve (for instance, with the introduction of improved shielding, filters and algorithms against electromagnetic interference). In this review, we discuss the various interactions that might occur between the two most common therapies in cardiac electrophysiology, and strategies for preventing adverse outcomes.

### Potential interactions between cardiac implantable devices and catheter ablation

Various interactions have been reported between catheter ablation and CIEDs, depending on how close the contact between them is, and the type of energy that is used. While radiofrequency (RF) and cryotherapy are currently the two main forms of energy used during catheter ablation, RF is by far the most common ablation modality.^4^ As such, potential interactions between ablation catheters and CIEDs may include (1) the effects of RF current (typically 500 to 1,000 kHz) on CIED pulse generators, including electromagnetic interference (EMI) oversensing and the resulting inappropriate sensing, as well as pacing and/or delivery of therapies in the form of inappropriate anti-tachycardia pacing and defibrillation shocks for ICDs; (2) transvenous lead dislodgment because of catheter manipulation; and (3) alterations in pacing, sensing or impedance parameters because of ablation completed in close vicinity or in direct contact with the lead.

### 1) Effects on the pulse generator

The majority of conventional pacemaker pulse generators are not adversely affected by exposure to RF energy during ablation, and those that are affected are done so transiently and often exhibit sensing-related issues.^6,7^

The majority of the effects during RF energy application are related to EMI, and may include: noise reversion mode with asynchronous pacing, oversensing of EMI with rapid tracking, spurious arrhythmia detection, mode switch or pacing inhibition, inappropriate pacing and/or transient reset to elective replacement interval.^6,8^ If rate response is on, pacing at the upper sensor rate may occur, since EMI can alter intrathoracic impedance measurements.^9^ Fortunately, modern pulse generators and leads are less sensitive to EMI due to the development of improved shielding, and we typically do not see adverse interactions.^10^ It is also important to consider that programming devices to address asynchronous pacing during catheter ablation can be associated with a low but possible risk of ventricular fibrillation/tachycardia, because of the pacing on the T-wave that occurs during the vulnerable ventricular period and/or short-long-short sequence.^11,12^ Meanwhile, cryoablation has not been reported to cause interference with CIED pulse generator function.^13^

Robotic ablation with remote magnetic catheter navigation (RMN) (Niobe^®^ system, Stereotaxis, St. Louis, MO, USA) can be associated with EMI, since it uses magnetic fields to move an ablation catheter. One in vitro study tested 121 explanted pacemakers and defibrillators not connected to leads at maximal field strength and found that 5% of the devices exhibited transient changes (the pacemakers only), but could be reprogrammed after being removed from the magnetic field.^14^ RMN, however has not been shown to adversely impact CIED function *in vivo.*^15–17^

In the case of subcutaneous defibrillators, to date there have been no reports of significant interactions with catheter ablation, though its use in the United States is rapidly increasing.^18^ Since the pulse generator and lead system are entirely subcutaneous, the anticipated potential interaction is EMI. Therefore, ICD therapy should be disabled prior to delivering RF energy. Another novel technology that is being utilized is the leadless pacemaker. In a recent study, fie patients underwent leadless transcatheter pacemaker implantation followed by atrioventricular (AV) nodal RF ablation (for uncontrolled AF) without any reported interactions.^19^ This is the only study to date that has evaluated RF ablation in patients with leadless pacemakers.

### 2) Lead dislodgement

In addition to electronic interference, there is always a risk of mechanical micro-or macrodislodgment occurring in patients with implanted devices who are undergoing catheter ablation, especially among recently implanted leads. In a study that assessed the effects of RF during atrial fibrillation ablation among 86 patients with CIEDs, there were no direct RF-related adverse effects on lead function. However, atrial lead dislodgement occurred in two patients with newly implanted leads of less than 6 months.^20^ There is a possibility that lead dislodgment might become more common with the current trend of fluoroscopy-free ablation procedures.^21^ However, other clinical studies have failed to show lead dislodgement secondary to RF ablation.^22–24^ Overall though, the risk of mechanical dislodgment remains possible if enough traction is applied on the lead. To minimize this complication, it is recommended that the process of lead maturation be allowed to occur. Lead maturation is an inflammatory reaction that is produced following lead tip contact with endocardium that results in distal electrode encapsulation within the myocardial tissue.^25^ This process typically occurs 2–6 weeks after implantation, and results in a more stable lead with the electrode embedded in the cardiac tissue.^26^ However, it is generally accepted that there is an increased chance of lead dislodgement up to 3 months after implant.^27^ Therefore, we recommend allowing for a minimum of 6 weeks after CIED implantation, but ideally 3 months, to pass before an ablation procedure is performed.

### 3) Effects of ablation close to or in direct contact with a CIED lead

*Effect on lead/tissue interface.* In certain cases, myocardial thermal lesions may occur at the tip of the pacemaker and ICD leads from transmitted RF energy applied to the distal electrode.^27^ Therefore, tissue alterations at the lead/myocardial tissue interface may result in decreased sensing or an increase in capture threshold, either transiently or permanently. This hypothesis was tested *in vitro* by Dick et al.^28^ using an RF ablation catheter at 55 W applied for 60 s on four different pacing and defibrillating leads at different sites, starting 1 cm from the tip of the lead in a tissue bath. Voltage at the tip of the lead was measured during RF energy application, and the leads were then connected to a pacemaker pulse generator to test for a change in function before and after ablation. RF energy delivered less than 1 cm from the electrode tip resulted in a significant current, which may be associated with tissue damage at the cardiac tissue-lead interface. An *in vivo* study in dogs reported that RF ablation about 1 cm from the tip of the pacing lead resulted in transient oversensing and inhibition of pacing output.^29^ Meanwhile, delivering RF lesions 4 cm or more from the lead did not result in lead malfunction.^29^ However, a prospective study showed that atrial ablation can be performed relatively safely in patients with pacemakers and defibrillators (although one patient in the study population had transient increase in atrial lead capture threshold and another had a decrease in atrial sensing after ablation).^30^ Therefore, the authors recommend delivering lesions at least 2 cm away from the distal pacing electrode. The same study showed that a higher number of linear lesions was required to achieve success, probably because of the challenge of delivering effective ablation lesions within the close vicinity of a CIED lead. Practically, it is not always possible to stay away from leads while applying RF energy, since critical areas for arrhythmia initiation or maintenance are sometimes located in the close vicinity of a CIED lead. In attempt to have better catheter control while ablating near a CIED lead, the use of a long sheath may be helpful in stabilizing the ablation catheter to avoid contact between the catheter and the lead.^23^ Another elegant method is to use a snare to gently remove the lead away from the ablation catheter site.^31^ Overall, ablating within 1 cm of the lead tip is not recommended, but it is safe for the lead-tissue interface if the catheter is more than 4 cm away from the tip.

*Effects on lead insulation.* Society guidelines and CIED manufacturers recommend against using RF energy in close proximity or in direct contact with transvenous leads. Lead insulation defect is the most common reason (56%) for industry-wide ICD lead failure.^32^ It has indeed already been demonstrated that RF-based electrocautery, which uses a higher amount of energy/power than catheter ablation, can create insulation damage, especially in polyurethane and copolymer leads (Optim™, St. Jude Medical, St. Paul, MN, USA) because of their lower thermal stability.^33^ Recently, RF energy application through commonly used ablation catheters was studied *in vitro* on various commercially-available transvenous CIED leads at maximum power, with irrigated tip catheters, non-irrigated tip catheters, and cryoablation.^34^ In this study, the transvenous leads tested included pacemaker, left ventricular and defibrillatory leads of three main commercially used insulation materials (i.e. silicone, polyurethane, and co-polymer). Interestingly, no significant effect was observed on the outer insulation or on lead functionality. This absence of lead damage can be explained by the fact that the temperature achieved with catheter-based RF energy is much lower than that achieved with surgical electrocautery. Generally, RF energy used for cardiac ablation is applied as a continuous, unmodulated sine wave at low voltage, whereas that used for electrocautery is high voltage with a long duty cycle that is designed to promote arcing or coagulum formation.^35^ Therefore, the temperature achieved by the RF ablation catheter (up to 106.1°C in the study) is much lower than that achieved by the RF electrocautery cut mode, and is much lower than the melting point of polyurethane (185–225°C) or silicone (has no specific melting point). In addition, the cooling effect of circulating blood flow confers a protective mechanism by helping to reduce the temperature when RF ablation energy is applied on or close to transvenous leads.

*Effects on defibrillator coils.* RF catheter ablation has been found to be associated with lead malfunction in two studies through a possible effect on defibrillator coils. A study of RF application for AV junction ablation among 59 patients with pre-existing pacemaker or defibrillator implants resulted in a rise in pacing threshold, requiring ventricular lead revision, particularly among the 13 patients with defibrillator leads. This led to lead revision among two out of 13 (15%) patients with defibrillators and two out of 46 (4%) with pacemakers.^36^ To date, the mechanism resulting in lead revisions in this study still remains unclear. Hypotheses include the role of the distal ICD coil in conducting some of the dispersed RF energy during ablation, or a possible lead micro-dislodgment, since macrodislodgment was not reported in this study. In a second study, RF has been reported to result in transient impedance changes in a defibrillator coil, but without longterm sequela.

### Peri-ablation protocol in patients with cardiac implantable devices

**Figure 3:**
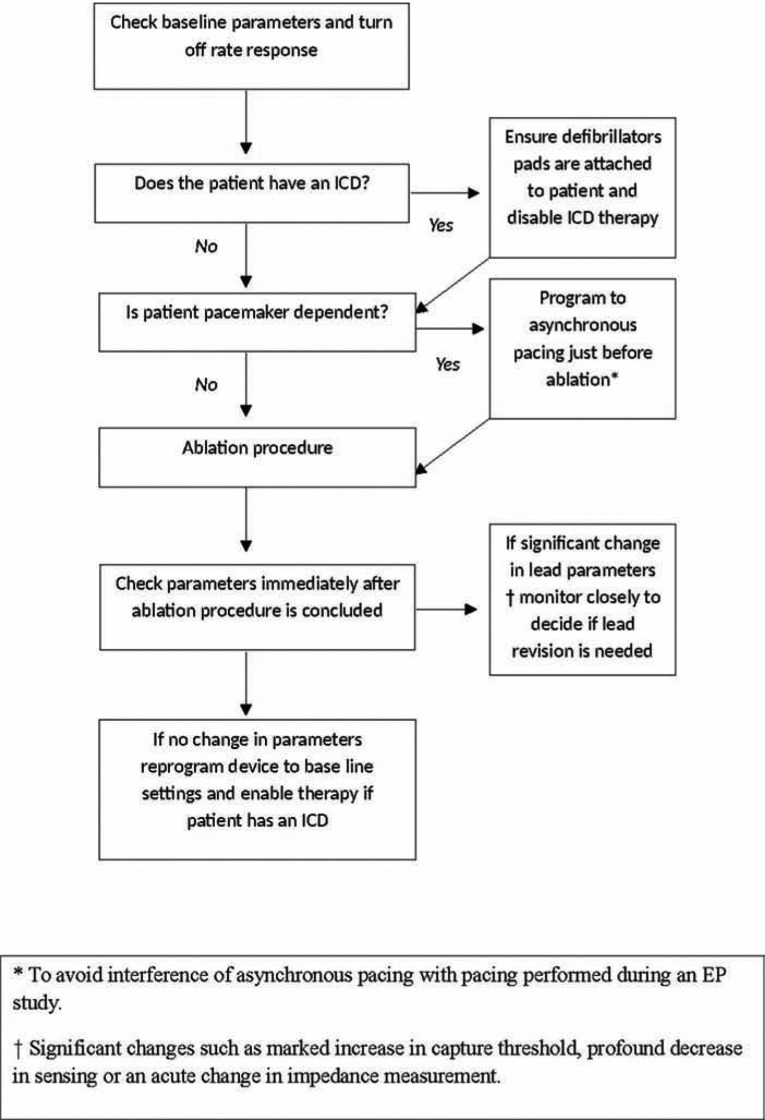
algorithm for managing permanent pacemakers and implantable defibrillators before and after a catheter ablation procedure.

At our institution, we use the following protocol before starting catheter ablation in a patient with a CIED: (1) turn rate response off (depending on type of rate response and manufacturer); (2) program the device to DOO or VOO mode if the patient is pacemaker-dependent; and (3) disable therapy (i.e. ventricular tachycardia or ventricular fibrillation detection is turned off) in a patient with an ICD. The different steps followed pre-ablation and post-ablation among patients with CIEDs in our institution are presented in **Figure 3**.

## Conclusion

Various interactions may occur between catheter ablation and CIEDs. It is advised, as a precautionary measure, to program the patient’s CIED prior to ablation to avoid defibrillator therapy and oversensing. In addition, though direct contact of the ablation catheter with the lead has not been proven to be deleterious, contact with the distal electrode of the lead should be avoided in order to prevent potential tip/tissue interface injury. Caution is also recommended while manipulating the catheter to avoid lead dislodgement. Overall, it is generally safe to perform catheter ablation in patients with CIEDs while using the aforementioned precautionary measures.
